# Inhibition of Mycobacterium-RmlA by Molecular Modeling, Dynamics Simulation, and Docking

**DOI:** 10.1155/2016/9841250

**Published:** 2016-02-14

**Authors:** N. Harathi, Madhusudana Pulaganti, C. M. Anuradha, Suresh Kumar Chitta

**Affiliations:** ^1^Department of Biochemistry, Sri Krishnadevaraya University, Anantapur 515 003, India; ^2^Department of Biotechnology, Sri Krishnadevaraya University, Anantapur 515 003, India

## Abstract

The increasing resistance to anti-tb drugs has enforced strategies for finding new drug targets against* Mycobacterium tuberculosis* (Mtb). In recent years enzymes associated with the rhamnose pathway in Mtb have attracted attention as drug targets. The present work is on *α*-D-glucose-1-phosphate thymidylyltransferase (RmlA), the first enzyme involved in the biosynthesis of L-rhamnose, of Mtb cell wall. This study aims to derive a 3D structure of RmlA by using a comparative modeling approach. Structural refinement and energy minimization of the built model have been done with molecular dynamics. The reliability assessment of the built model was carried out with various protein checking tools such as Procheck, Whatif, ProsA, Errat, and Verify 3D. The obtained model investigates the relation between the structure and function. Molecular docking interactions of Mtb-RmlA with modified EMB (ethambutol) ligands and natural substrate have revealed specific key residues Arg13, Lys23, Asn109, and Thr223 which play an important role in ligand binding and selection. Compared to all EMB ligands, EMB-1 has shown better interaction with Mtb-RmlA model. The information thus discussed above will be useful for the rational design of safe and effective inhibitors specific to RmlA enzyme pertaining to the treatment of tuberculosis.

## 1. Introduction

Tuberculosis (TB) caused by* Mycobacterium tuberculosis* (Mtb) remains one of the world's greatest causes of mortality and morbidity with 8 million new infections and 2 million deaths per year [1]. Mtb has managed remarkably to infect an estimated one-third of the world's population [2, 3]. The emergence of multidrug-resistant (MDR) Mtb strains [4], coupled with the increasing overlap of the AIDS [5, 6], variable efficacy of Bacille-Calmette-Guerin (BCG) vaccine [7], lack of patient compliance with chemotherapy, and TB pandemics, has brought TB to the forefront as a major worldwide health concern. It has been estimated that 31% of AIDS cases can be attributed to TB in the African region [8, 9]. The deadliest disease is required to be treated with advanced technology. Therefore, new approaches to the treatment of tuberculosis are needed.

**Figure 1:**
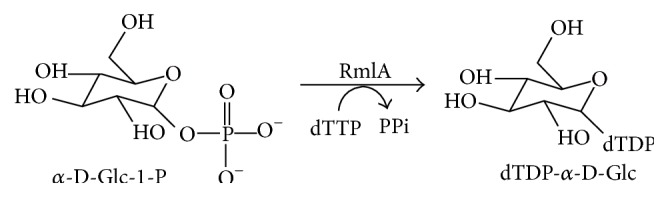
Diagrammatic representation of reaction catalyzed by RmlA from Mtb.

For this new emerging field,* in silico* drug design has offered enormous benefits for the development of effective drugs against TB. In this context, we have chosen the enzymes involved in L-rhamnose synthesis of Mtb, which plays an essential structural role in the cell wall formation. Mycobacterial cell wall is essential for viability [10]; it represents a very attractive target [11] for new antibacterial agents. The cell wall core consists of three interconnected macromolecules. The outermost mycolic acids [12, 13] are 70 to 90 carbon-containing branched fatty acids that are esterified to the middle component, arabinogalactan (AG), a polymer composed primarily of D-galactofuranosyl and D-arabinofuranosyl residues. AG is connected via a linker disaccharide, *α*-L-rhamnosyl-(1→3)-*α*-D-N-acetyl-glucosaminosyl-1-phosphate, to the sixth position of a muramic acid residue of the peptidoglycan [14], which is the outermost of the three cell wall core macromolecules. Moreover, rhamnose residue, a sugar that was not found in humans, plays a crucial structural role in the attachment of AG to the peptidoglycan. The precursor of L-rhamnose is dTDP-L-rhamnose (dTDP-Rha) which functions as a Rha donor for the linker region in mycobacteria [15] in the presence of rhamnosyl transferase enzyme [16]. The pathway of dTDP-Rha biosynthesis has been studied extensively in Gram-negative bacteria [17]. dTDP-Rha is synthesized from deoxy-thymidine triphosphate (dTTP) and *α*-D-glucose-1-phosphate (*α*-D-Glc-1-P) by a single pathway which involves a series of four enzymes, that is, RmlA, RmlB, RmlC, and RmlD, encoding *α*-D-Glc-1-P thymidylyltransferase [18], dTDP-D-Glc 4,6-dehydratase [19], dTDP-4-keto-6-deoxy-D-Glc 3,5 epimerase [20], and dTDP-rhamnose reductase [21]. Inhibition of biosynthesis of L-rhamnose residue would be lethal to bacteria by making a linker disaccharide unit impossible, which results in the disruption of structural integrity of the cell wall and in turn leads to cell lysis. Availability of complete genome sequence of Mtb H37Rv [22] strain greatly aids in the identification of the enzymes involved in dTDP-Rha synthesis and helps the conception of new prophylactic and therapeutic interventions. Significantly this pathway does not exist in mammals and all four enzymes therefore represent potential therapeutic targets. In this aspect, we have chosen first enzyme, that is, *α*-D-Glc-1-P thymidylyltransferase (RmlA) (2.7.7.24), in the dTDP-Rha pathway of Mtb. It catalyzes the reaction that combines dTTP with *α*-D-Glc-1-P to yield dTDP-glucose and pyrophosphate (PPi) (shown in Figure 1). This reaction constitutes the first step in the synthesis of L-rhamnose, a component of the cell walls of both Gram-negative bacteria and Gram-positive bacteria [23].

Due to the unavailability of crystal structure of Mtb-RmlA, we have employed* in silico* approaches to resolve and characterize the structure of this important enzyme by molecular modeling and simulation techniques. Global and local accuracy of the predicted model was assessed by various assessment programs. With the aim to build novel inhibitors for Mtb-RmlA model, docking studies are done with series of ethambutol (EMB) derived ligands. Results of ligand interactions have revealed specific residues in the binding domain of Mtb-RmlA. This information could be exploited for future designing of more effective inhibitors for Mtb-RmlA enzyme. Mtb-RmlA model is specific for Mycobacterium-RmlA, which is novel drug target for drug designing.

## 2. Methodology

The study was conducted by the author in the Department of Biochemistry using Intel Pentium IV 3.4 MHz, AMD Althon 64 bits dual processor with 4 GB RAM, and video graphics card. Molecular modeling tasks were performed with Modeller 9v1; MD simulations were analyzed with Gromacs 3.2.1; docking calculations were performed with AutoDock 3.0; if not otherwise stated, default settings were used during all calculations.

### 2.1. Sequence Alignment and Molecular Model Generation

Mtb-RmlA amino acid sequence (UniProtKB-P9WH13) was obtained from National Center for Biotechnology Information (NCBI) [24] in FASTA format [25]. Homologous entries for Mtb-RmlA sequence were obtained from Protein Data Bank [26] using Blastp (Basic Local Alignment Search Tool) [27] at NCBI. All the derived entries were aligned with Mtb-RmlA sequence using a multiple sequence alignment tool at ClustalX 1.83 [28], which reveals functionally important conserved residues in all RmlA families. Based on this sequence alignment, tertiary structure of RmlA enzyme was built using Modeller 9v1 [29] software by satisfaction of spatial restrains [30]. The program was carried out using standard parameter set and databases. Many runs of model building were carried out to obtain the most reasonable model and subsequently the best model (with the low RMS value of superposition using Swiss-pdb viewer [31]) was subjected to further analysis. To remove steric clashes arising from nonbonded interactions and to correct the bad geometry in RmlA model and to achieve a good starting structure, refinement was done by energy minimization (EM) and molecular dynamic (MD) simulations using Gromacs 3.2.1 package [32] and in particular 43A1 (Gromacs 96) force field.

### 2.2. Molecular Dynamics Simulation

MD is a computationally demanding procedural challenge for which several well-known solutions exist. We find Gromacs to be of outstanding interest because the software is well tuned for common hardware and advanced algorithmic optimizations, allowed for remarkable computational speed. It solves Newton's equations of motion for a given system over a specified period of time. Best Mtb-RmlA model obtained from homology modeling was immersed in a solvent octahedral box of SPC (simple point charge) water model [33, 34] and ions (Na^+^ and Cl^−^) were added to neutralize the system. Using the MD protocol, all hydrogen atoms, ions, and water molecules were subjected to 50 rounds of energy minimization using steepest descent algorithm [35] till an energy gradient was reached. This dynamic allows the equilibration of the solvent around the protein residues and all protein atoms had their positions restrained. Mtb-RmlA model was subjected to a full MD simulation of 5000 ps at 300 K (temperature of the system was increased in 5 steps 50–100, 100–150, 150–200, 200–250, and 250–300) with no restrictions using 2fs of integration time. All protein covalent bonds were constrained using LINCS [36] to maintain constant bond length and the Settle algorithm was used to constrain the intramolecular water bonds to their equilibrium length [37]. Coordinates and energy terms (total, kinetic, and potential for the whole system and electrostatic, distance-dependent, distance-independent reaction force field) were saved for each ps. The changes in structural conformation have been monitored in terms of RMSD, energy data, and RMSF. The stabilization was assessed by graphic visualization.

### 2.3. Structural Validation of the Homology Model

To predict a good quality model, it is very important that appropriate steps are built into the process to assess the quality of the model [38]. Stereochemical properties were evaluated through Procheck [39]. Backbone conformation was evaluated by investigating psi/phi angles in Ramachandran plot using Procheck [39]. Bond lengths, bond angles, *Z*-scores, and energy plots were evaluated by Whatif [40] and ProsA [41]. Furthermore, the Mtb-RmlA model was also submitted to the Verify 3D [42, 43], a structure evaluation server in order to check the compatibility of each residue with the current 3D model. The compatibility between the amino acid side chains of each amino acid in the model is a validation criterion. Overall quality factor for nonbonded interactions of Mtb-RmlA was checked by Errat [44]. The 3D model that scores high in all these evaluation tests is regarded as the most satisfactory model of Mtb-RmlA. Secondary structural conformations of Mtb-RmlA model were predicted by Pdbsum [45] online server, which provides complete data about the helices, beta sheets, and turns present in the structure. The software Pymol [46] is a flexible extensible package for molecular visualization which is used to generate clear and attractive representation of atomic data. Motif scan server was used for identification of domains in the built model [47]. The developed Mtb-RmlA model was submitted to Protein Model Database (PMDB) [48], which collects the 3D models obtained by structure prediction methods.

### 2.4. Docking Studies of Mtb-RmlA

To investigate the most probable binding sites in Mtb-RmlA model and further to check its suitability for use in structure based drug design, docking studies were done with AutoDock 3.0 [49] program. Several front line drugs are known to target the essential components of the Mtb cell wall. Among those, in the present work we have chosen an effective drug EMB (ethambutol) [50, 51] which inhibits the attachment of the peptidoglycan layer to mycolic acid layer by inhibiting the formation of Arabian region of arabinogalactan and finally effects the growth of mycobacteria. Hence, in the current study, a library of 50 ligand molecules was constructed based on the seed structure of EMB and implementing structural manipulations and optimizations on it by ChemDraw (Cambridgesoft Inc.) [52]. The generated new EMB ligands were tested for Lipinski's rule of five, using Molinspiration server [53] for their acceptable physical properties and chemical functionalities. To the screened ligands, atomic partial charges were added using Prodrg server [54]. Preparation of Mtb-RmlA model for docking involves the addition of polar hydrogens, using the hydrogens module in AutoDockTools (ADT) for Mtb-RmlA; after that Kollman united atom partial charges were assigned [55]. The proteins were treated as rigid bodies during docking simulations but all the torsional bonds in ligands were set free to perform flexible docking. To find suitable binding position for a ligand on a given protein, grid maps were calculated with AutoGrid. The grid points in *x*, *y*, and *z* axes were set to 60 × 60 × 60 Å with grid spacing of 0.375 Å. For flexible docking, Lamarckian genetic algorithm [56] was selected. The maximum number of energy evaluations and number of energy iterations were set to 2,000,000 and 27,000, for an initial population of 300 randomly placed individuals. The mutation rate, crossover rate, and elitism value were 0.02, 0.80, and 1, respectively. For each ligand, a docking experiment consisting of 100 simulations was performed. Docking evaluations are based on free energy of binding, lowest docked energy, and cluster RMSD values, and ligand molecules were then ranked in the order of increasing docking energies. Substrate docking with natural substrate: that is, Glc-1-P was also performed on Mtb-RmlA model with the same parameters. The ligand-receptor complexes were analyzed using Pymol program [46]. Binding energy is one which is disassembling a whole system into separate parts. A bound system typically has a lower potential energy than the sum of its constituent parts; this is what keeps the system together. Often this means that energy is released upon the creation of a bound state. Docked energy is the interaction energy between protein and ligand only (interface_delta); this is the score difference between the components together and the components pulled apart by 500 Å.

## 3. Results

### 3.1. Sequence Analysis and Homology Modeling of Mtb-RmlA

**Figure 2:**
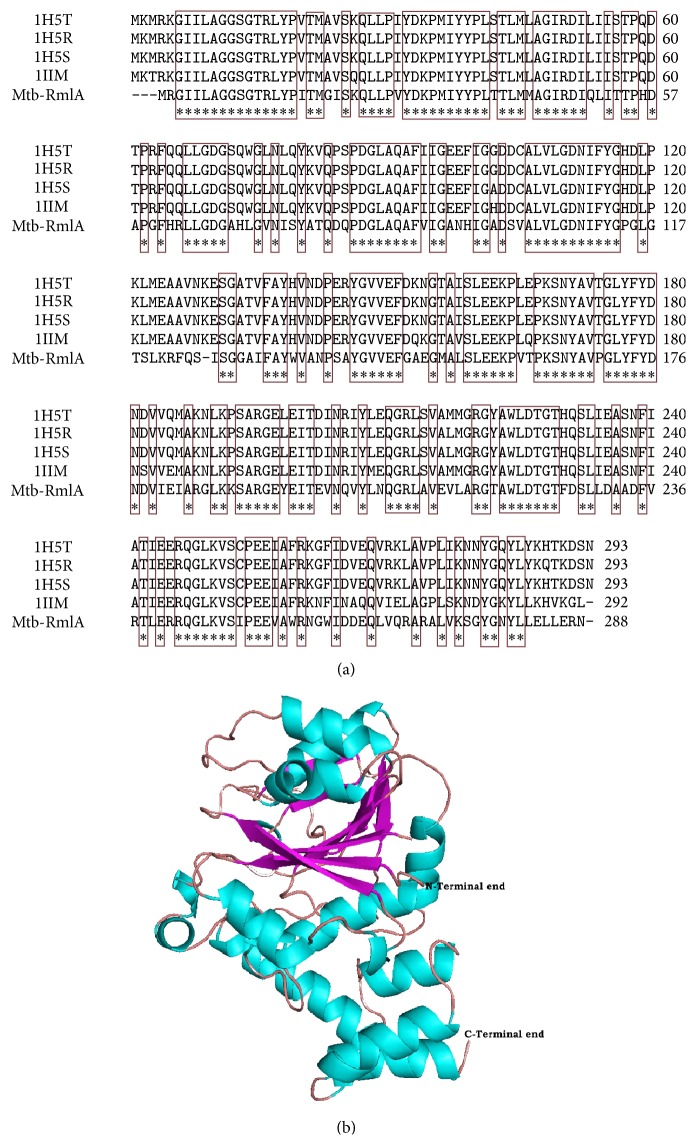
(a) Multiple sequence alignment of Mtb-RmlA and the templates 1H5T, 1H5R, 1H5S, and 1IIM. Dashes represent insertions and deletions. Highly conserved residues are represented in rectangular boxes. (b) The developed 3D model of Mtb-RmlA shown in cartoon representation with helices in cyan, sheets in magenta, and turns in wheat.

Mtb-RmlA amino acid sequence containing 288 amino acids was obtained from NCBI in FASTA format with UniProtKB-P9WH13. Crystal structures from* Ecoli* (Pdb ids: 1H5T, 1H5R, and 1H5S) [57] and* Salmonella enterica* (Pdb ids: 1IIM) [58] (Table 1), exhibiting sequence homology with Mtb-RmlA, were obtained by Blastp analysis and thus chosen as templates for developing the Mtb-RmlA model. The sequence identities between Mtb-RmlA and templates 1H5T, 1H5R, 1H5S, and 1IIM were 62%, 62%, 63%, and 60% (Table 1), respectively. High level of sequence identity could produce a more accurate alignment between the target sequence and homologues. The sequence alignment performed using ClustalX [28] for homology modeling is shown in Figure 2(a). The alignment was manually refined and final results show that five residues are deleted in the entire structure, in which three are at N-terminal end and one at middle (position 126) and remaining ones at the end of the chain. Figure 2(a) reveals that the residues involved in binding of various feedback inhibitors in templates (Leu9, Gly11, Gln12, Gln83, Pro86, Asp87, Gly88, Asp111, Tyr115, Gly116, His 111, Asp118, Gly219, Gly221, and Ser 252) were conserved in Mtb-RmlA.

The appropriate template was chosen based on sequence similarity, residue completeness, and crystal resolution. To elucidate the 3D structural features of Mtb-RmlA we used comparative modeling analysis and in particular Modeller 9v1 program. This program uses the spatial constraints determined from the crystal structures of* Ecoli* (Pdb ids: 1H5T, 1H5S, and 1H5R) [57] and* Salmonella enterica* (Pdb ids: 1IIM) [58] (Table 1) to build a 3D model of Mtb-RmlA (Figure 2(b)). A total of 100 models of Mtb-RmlA were generated and among them the one having lowest root mean square deviation (RMSD) value when superposed onto the templates (1H5T, 1H5S, 1H5R, and 1IIM) was selected for further analysis [31]. The tertiary structure of Mtb-RmlA shows close resemblance to templates with backbone RMS values between Mtb-RmlA-1H5T, Mtb-1H5R, Mtb-1H5S, and Mtb-1IIM which are 0.60 Å, 0.57 Å, 0.65 Å, and 0.61 Å, respectively (supporting data in Supplementary Material available online at http://dx.doi.org/10.1155/2016/9841250). The low RMSD values for backbone superposition reflect the high structural conservation of this complex through evolution making a good system for homology modeling.

### 3.2. Analysis of the MD Simulation

**Figure 3:**
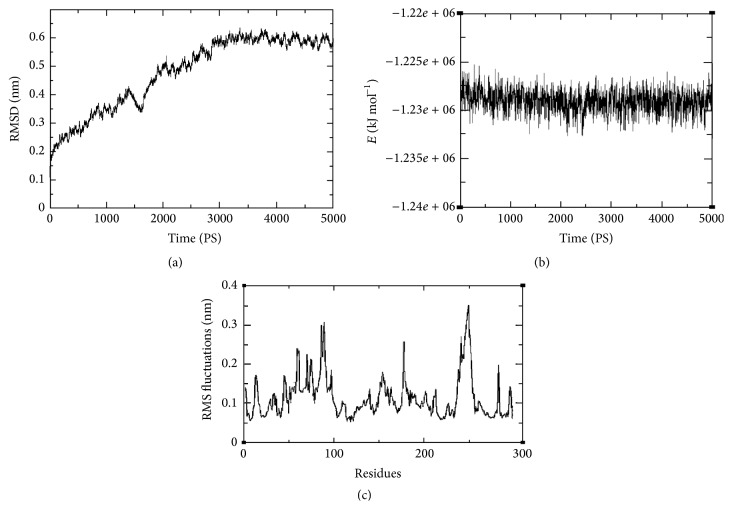
(a) Graphical representation of RMSD of back bone carbons from starting structure of Mtb-RmlA as a function of time. (b) The potential energy curve of the system during the MD simulation for Mtb-RmlA. (c) RMS fluctuations for the total protein of Mtb-RmlA.

The structural stability of the predicted Mtb-RmlA model was tested by MD simulations. The trajectories were stable during the whole production part of 5000 ps MD simulation run. The trajectory stability was monitored and was confirmed by the analysis of backbone RMSD (Figure 3(a)) and the total energy (Figure 3(b)) as a function of time for the Mtb-RmlA. RMSD measures the accuracy, whereas dynamic fluctuations (RMSF) of proteins around their average conformations are an important indicator of many biological processes such as enzyme activity, molecular recognition, and complex formations [59]. A rise in the RMSD values in the first 3000 ps of simulation is observed for Mtb-RmlA in Figure 3(a) and then reached stable in the following simulation time. A rise in the value in the first 3000 ps is attributable to the relaxation motion of the protein or inaccuracy in the force field. The average RMSD for the Mtb-RmlA model when measured from 5000 ps was found to be ~0.6155708 nm. Total energy (KJ mol^−1^) (Figure 3(b)) was found to be stable throughout the simulation time. The total RMSF (peptide backbone + side chains) was showed for the developed model in Figure 3(c). The graph showed that the residues at N-terminal regions have lower RMSF values. In a typical RMSF pattern, a low RMSF value indicates the well-structured regions while the high values indicate the loosely structured regions or domains terminal [60]. It was found that throughout dynamics simulations maximum fluctuations were passed ~0.15 nm for total protein. These fluctuations are due to the presence of network of hydrogen bonding stabilizing the secondary structures,* that is*, *α*-helix and *β*-sheet. Very few fluctuations have exceeded 0.3 nm and even less fluctuations overpassed 0.35 nm for total protein.

### 3.3. Validation of Homology Model

**Figure 4:**
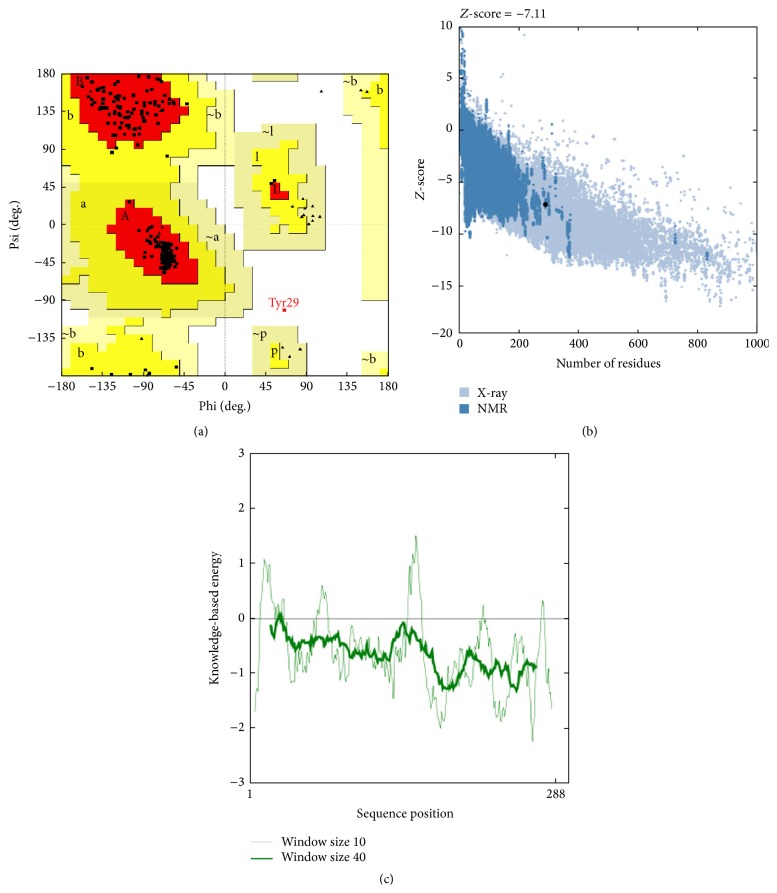
(a) Ramachandran plot for predicted Mtb-RmlA model. (b) ProsA-web *Z*-scores of all protein chains in PDB determined by X-ray crystallography (light blue) and NMR spectroscopy (dark blue) with respect to their length. The *Z*-score of Mtb-RmlA was present in that range represented in large black dot. (c) Energy plot for the predicted Mtb-RmlA model.

The overall stereochemistry of each residue in Mtb-RmlA model was checked using Ramachandran plot calculations computed with Procheck Program [39]. The analysis reveals that 99.6% residues were positioned in favored and allowed regions of the Ramachandran plot (Figure 4(a)). In comparison with templates, the homology model had a similar Ramachandran plot with 0.4% residues in disallowed regions (Table 2). The goodness factor (*G*-factor) provides a measure of how “normal,” or alternatively how “unusual,” a given stereo chemical propriety is. The *G*-factor of Mtb-RmlA was found to be zero (acceptable values of the *G*-factor in Procheck are between 0 and −0.5, with the best models displaying values close to zero) which indicates a good quality of the model. Standard bond lengths and bond angles of Mtb-RmlA model were determined by using Whatif web interface [40]. The analysis revealed RMS *Z*-scores for bond lengths and bond angles as 0.910 and 1.185, respectively. The values are close to 1 and also within the limits of templates (Table 2).

**Figure 5:**
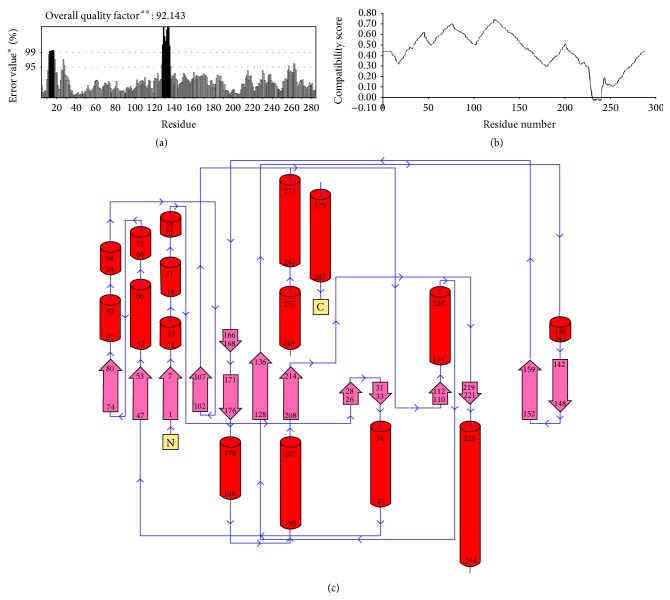
(a) Errat score for the Mtb-RmlA model. (b) The 3D profile verified results of predicted Mtb-RmlA model. The residues with positive compatibility score are reasonably folded. (c) Diagrammatic presentation of Mtb-RmlA model demonstrating various secondary structural elements. “*∗*” represents the conserved regions and “*∗∗*” represents the semiconserved regions.

ProsA-web was used to check the three-dimensional model of Mtb-RmlA for potential errors [41]. It displaces *Z*-scores and energy plots that highlight potential problems in protein structure. The *Z*-score indicates overall model quality and measures the deviation of the total energy of the structure with respect to an energy distribution derived from random conformations. As shown in Figure 4(b), the *Z*-score for Mtb-RmlA is −7.11 which is in the range of native conformations of crystal structures (Table 2). ProsA-web analysis had showed that overall the residue energies of the Mtb-RmlA model (Figure 4(c)) remain negative for almost all amino acid residues except for some peaks in the starting region, indicating the acceptability of the predicted model. Overall quality factors of nonbonded interactions between different atom types of Mtb-RmlA were measured by using Errat plots [44]. The normal accepted range is >50 for a high quality model [44]. In the current case, Errat showed an overall quality factor for Mtb-RmlA as 92.143 (Figure 5(a)), well within the range of a high quality model; in the mean time the Errat score for template 1H5T is 94.286, for 1H5S is 95, for 1H5R is 98.925, and for 1IIM is 98.571 (Table 2). In Errat plot, errors in model building (aa10–20 and 120–140) lead to more randomized distributions of the different atom types, which can be distinguished from correct distributions by statistical methods. Atoms are classified in one of three categories: carbon (C), nitrogen (N), and oxygen (O). This leads to six different combinations of pairwise noncovalently bonded interactions (CC, CN, CO, NN, NO, and OO) [44]. The final structure was also assessed by Verify 3D [42, 43].  Figure 5(b) represents the Verify 3D graph of the predicted Mtb-RmlA. A score above zero for a given residue corresponds to acceptable side chain environment. From Figure 5(b), it is observed that almost all residues are reasonable, but only a few residues are variable (Asp231-Glu240) and are built poorly. Regarding the main chain properties of the modeled enzyme, the careful examination of the checking results was performed at the Procheck [39]. The results show that (Table 3) the Mtb-RmlA model lies within allowed region for all six parameters checked. Side chain parameters [39] of Mtb-RmlA model were obtained from Procheck, which reveal that the chi-gauche minus standard deviation, trans standard deviation, gauche plus standard deviation, chi pooled standard deviation, and chi-2 trans deviation standard deviation values (Table 3) are within the expected range.

**Figure 6:**
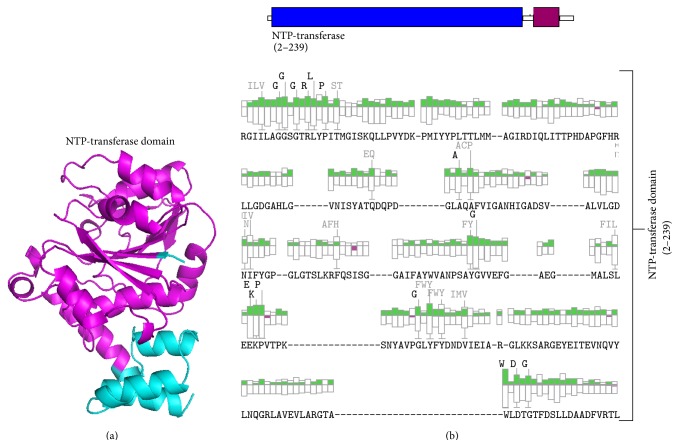
(a) Organization of NTP-transferase domain in Mtb-RmlA model with Scansite server. (b) Organization of various domains in NTP-transferase domain (2–239).

The secondary structure analysis of Mtb-RmlA model with Pdbsum [45], a secondary structure prediction server, reveals that 61 (21.2%) residues were in *β*-strands, 105 (36.4%) residues were in *α*-helices, 12 residues (4.2%) were in 3–10 helices, and 110 (38.2%) residues were in other conformations (Figure 5(c)). In order to investigate the organization of various domains in the developed model of Mtb-RmlA model, it was subjected to Scansite server [47]. It was reported that Mtb-RmlA has N-terminal or NTP-transferase domain (2-239) [61] (Figures 6(a) and 6(b)). This domain occupies a major portion of the Mtb-RmlA model and plays an important role in binding to inhibitors. The function of this domain is to transfer the nucleotides to the phosphosugars. The enzyme family includes alpha-D-Glc-1-P cytidylyltransferase, mannose-1-phosphate guanylyltransferase, and Glc-1-P thymidylyl transferase. The products are activated sugars that are precursors for synthesis of lipopolysaccharides, glycolipids, and polysaccharides.

In brief, the geometric quality of the backbone conformation, the residue interaction, residue contact, energy profile, and nonbonded interactions of the structure are all well within the limits established for reliable structures and provide strong confidence of the homology model. Passing all tests by predicted model suggests that an adequate model for Mtb-RmlA is obtained to characterize protein-substrate and protein-ligand interactions and to investigate the relation between the structure and function. With all these evaluations the predicted Mtb-RmlA model was submitted to PMDB and it has accepted the model with less than 3% stereochemical check failures. PMDB ID for the developed Mtb-RmlA model was PM0076036.

### 3.4. Design, Validation (Drug), and Docking Studies of Mtb-RmlA Inhibitors

**Figure 7:**
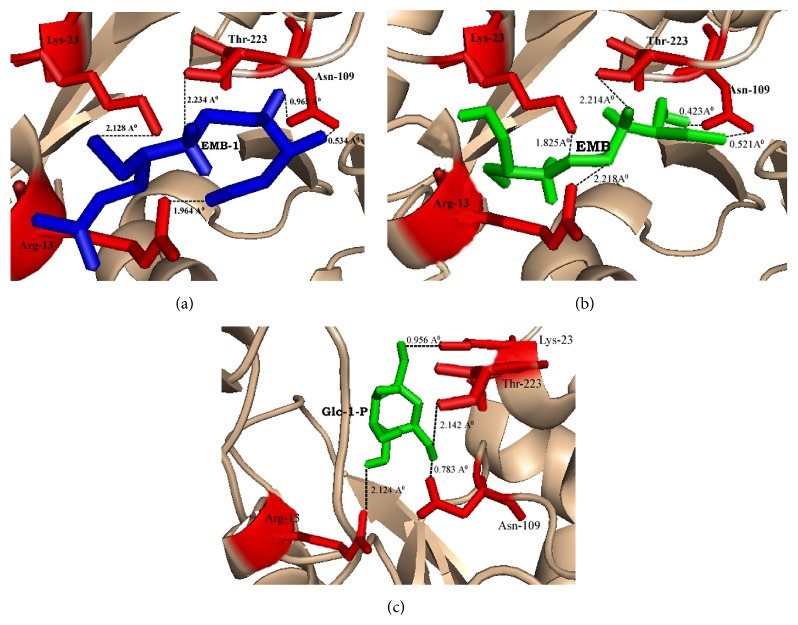
(a) Interaction of EMB-1 ligand with active site amino acids of Mtb-RmlA model. (b) Interaction of EMB ligand with active site amino acids of Mtb-RmlA model. (c) Interaction of natural substrate (Glc-1-P) with active site amino acids of Mtb-RmlA model. Built model of Mtb-RmlA is represented in cartoon. Ligands and the residues interacting with ligands are represented by sticks.

To gain insight into the binding conformations of designed ligands and Mtb-RmlA model, we followed molecular docking protocol as described in Materials and Methods. Using the parent molecule of EMB (ethambutol), a library of 35 (Supplementary table) EMB ligands was drawn and optimized with the aid of ChemDraw [52]. All new compounds were tested for their ability to follow ADME rules. Among the 50, 5 lead molecules satisfying rule of five with zero violations were chosen for docking on Mtb-RmlA model. AutoDock 3.0 [49] and its graphical front-end AutoDock Tools (ADT) were used to perform docking calculations. Analysis of docking (dlg) file of each EMB molecule gives 15 best simulations among the 50, which were observed through ADT. The top simulations for each ligand molecule showed interactions with predicted active site amino acids such as Arg13, Lys23, Asn109, and Thr223 of Mtb-RmlA model. Ligand molecules form hydrogen bond with Arg13 gaunidno group, Lys23 amino group, Asn109 amide group, and Thr223 hydroxyl group. During all these interactions hydrogen bond is found to play a vital role between ligands and active site residues of RmlA. In most cases hydrogen bond decides the binding strength and location of the ligand, whereas hydrophobic interactions of certain groups affect the inhibition specialty to a larger extent. All the ligand molecules showed good binding conformations with the Mtb-RmlA model. The rank of each ligand molecule was based on free energy of binding, lowest docked energy, and calculated RMSD values (Table 4). Among all docked ligands, EMB-1(2-[2-(1-methoxymethyl-propylamino)-ethylamino]-propan-l-ol) (Figure 7(a)) had shown best predicted binding energy of −6.04 kcal/mol, docked energy of −8.88 kcal/mol, and RMSD of 0.13 Å to the Mtb-RmlA model (Table 4). Following the same parameters, docking of Mtb-RmlA is also performed with parent EMB (Figure 7(b)). It has shown less docked energy of −7.69 kcal/mol, binding energy of −4.9 kcal/mol, and RMSD of 1.54 Å, compared to all EMB ligands (Table 4). To confirm the mode of binding of designed ligand molecules, natural substrate docking with Glc-1-P was performed on the Mtb-RmlA model with the same parameters. Natural substrate docking revealed that the amino acids Arg13, Lys23, Asn109, and Thr223 (Figure 7(c)) played vital role in binding the natural substrate. The binding free energy, docked energy, and RMSD of this complex were −6.01 kcal/mol, −8.85 kcal/mol, and 0.19 Å. In summary this detailed analysis helps to understand the binding modes of Mtb-RmlA model and its ligands and avoid obvious pitfalls in the detection of new ligands.

## 4. Conclusion

The present research work uses bioinformatics approach aimed to understand the Mtb-RmlA at molecular level. So an attempt has been made for* in silico* prediction for wet lab support in determination of three-dimensional structure of Mtb-RmlA through molecular modeling and simulation techniques. Since this pathway is not found in humans, this makes RmlA an attractive target for molecule inhibitors with the potential to have broad antibacterial activity. The average sequence identity between templates and Mtb-RmlA is ~61.75% which is more than a threshold value (30%) to predict the reliable structure with low RMS error. Multiple sequence alignment of Mtb-RmlA (Figure 2(a)) has revealed structurally important 166 conserved residues (shown in red color boxes) in all RmlA enzymes from different families, which play a vital role in the evolution of protein molecule. As there are less gaps and variations in sequence alignment of Mtb-RmlA, this indicates that model is straightforward to construct and structural difference in the model is limited to loops only. Among the 100 developed models the one having lowest RMS-superposition of carbon alpha and carbon backbone on the templates 1H5T, 1H5S, 1H5R, and 1IIM (0.60 Å, 0.57 Å, 0.65 Å, and 0.61 Å) (Figure 2(b)) was selected for further analysis, confirming that the model was satisfactory regarding the utilization of chosen templates for homology modeling process. By applying structural superposition and RMS evaluations, our model appears very similar to experimental one. The structural stability of the model was tested by MD simulations. MD Analysis shows that the total, kinetic, potential energies remained constant up to the end of the simulation. Overall shape and size of the molecule are remarkably stable till the end of 5000 ps of simulations and do not undergo any significant change. Thus, more relaxed and refined structure was finally produced which can be used for further analysis. As shown in Table 2 the homology model of Mtb-RmlA satisfies stereochemical restrains and passed all criteria carried out in Procheck, ProsA, and Whatif. Ramachandran plot analysis showed that 99.6% residues are in the most favored, additional, and generous regions. It is generally accepted that if 90% residues are in the allowed region, the quality of the model is evaluated as good and reliable. RMS *Z*-score values for bond lengths and angle parameters (Table 2) for the developed Mtb-RmlA model did not deviate significantly from the standard values and also within values typical of highly refined structures. The fact that the RMS *Z*-score values of bond distances and angles for the crystal structures are small might indicate that too strong constraints have been used in the original refinement of 1H5T, 1H5R, 1H5S, and 1IIM and there is no significant difference observed between the calculated values of the bond lengths and angles with that of known proteins for total residues. The interaction energy of each residue was checked by ProsA. The ProsA analysis of Mtb-RmlA model revealed that the residue energies including pair energy, combined energy, and surface energy are all negative and have similar tendency with the templates (Figures 4(b) and 4(c)). Thus, we conclude that Mtb-RmlA model had reached the energy criteria of ProsA. The compatibility score above zero in Verify 3D graph of Mtb-RmlA corresponds to the acceptable side chain environment. In the current case, Errat showed the overall quality factor 92.143 for the model, a result excepted for crystallographic models. The main chain properties of Mtb-RmlA model did not seem to contain considerable bad contacts, or *C*_*α*_ tetrahedron distortion, or buried unsatisfied H-bond donors and acceptors and also no distortions of the side chain torsion angles. Through this assessment and analysis process, we can conclude that the 3D structure of Mtb-RmlA constructed is reliable. Validity of the model is further assessed by docking studies. Docking results of Mtb-RmlA with natural substrate and designed ligands provide strong confidence about the homology model. It is obvious that this docked model would provide more detailed information and accuracy in its description of ligand binding with Mtb-RmlA model. Docking of EMB ligands and natural substrate to Mtb-RmlA model showed good* in vitro* inhibitory activity against Mtb-RmlA which are identified. All docked molecules showed hydrogen bonding with Arg13, Lys23, Asn109, and Thr223 amino acids of Mtb-RmlA. It is highly conceivable that these hydrogen bonding interactions play a vital role in the selection of potent and selective Mtb-RmlA inhibitors. Finally we concluded that valuable insight information into Mtb-RmlA model will help in understanding the mechanism action of Mtb-RmlA. Further, this work will guide us to design clinically significant anti-tb drugs against multidrug-resistant strains in less time as per pharmaceutical norms. The above research work will guide all researchers for further advance towards the treatment of this disease. This work also aims to prove that this disease is no longer incurable but the cure may be hidden in some other form.

## Supplementary Material

With the tertiary structure of Mtb-RmlA of templates retrieved from RCSB-PDB, they have backbone RMS values of Mtb-RmlA-1H5T, Mtb-1H5R, Mtb-1H5S, and Mtb-1IIM which are 0.60 Å, 0.57 Å, 0.65 Å, and 0.61 Å, respectively.

## Figures and Tables

**Table 1:** List of selected templates obtained from PDB for modeling Mtb-RmlA model.

Templates	Chain	Model	Number of residues in templates	Resolution Å	Percentage similarity	*R*-value
1H5T	A	X-ray	293	1.9	62	0.174
1H5S	D	X-ray	293	2.3	62	0.176
1H5R	B	X-ray	293	1.9	63	0.173
1IIM	A	X-ray	292	2.1	60	0.198

**Table 2:** Procheck values for the predicted Mtb-RmlA model and the template structures.

Ramachandran plot statistics	1H5T (Achain)	1H5S (Dchain)	1H5R (Bchain)	1IIM (A hain)	Mtb-RmlA
% amino acids in most favored regions	91.5%	89.9%	93.5%	92.3%	95.4%
% amino acids in additional allowed regions	8.1%	9.7%	6.1%	7.3%	4.2%
% amino acids in generously allowed regions	0.0%.	0.0%	0.0%	0.0%	0.0%
% amino acids in disallowed regions	0.4%	0.4%	0.4%	0.4%	0.4%
ProsA *Z*-score	−9.11	−9.15	−8.63	−8.93	−7.11
RMS *Z*-score					
Bond angles	0.934	0.890	0.894	0.709	1.185
Bond lengths	0.775	0.751	0.690	0.369	0.910
Errat score	94.286	95	98.925	98.571	92.143

**Table 3:** Main chain and side chain values for Mtb-RmlA obtained from Procheck.

Stereochemical parameter	Number of data points	Parameter value	Typical value	Band width	Number of band widths from mean
Main chain stereochemistry					
% of tag residues	241	95.4	83.8	10.0	1.2
Omega angle SD	286	3.6	6.0	3.0	−0.8
Bad contacts/100 residues	3	1.0	4.2	10.0	−0.3
Zeta angle SD	255	1.4	3.1	1.6	−1.1
H-bond energy SD	184	0.7	0.8	0.2	−0.4
Overall *G*-factor	288	0.0	−0.4	0.3	1.3
Side chain stereochemistry					
Chi-1 gauche minus st dev	29	7.3	18.1	6.5	−1.7
Chi-1 trans st dev	78	8.4	19.0	5.3	−2.0
Chi-1 gauche plus st dev	108	6.9	17.5	4.9	−2.2
Chi-1 pooled st dev	215	7.7	18.2	4.8	−2.2
Chi-2 trans st dev	63	8.3	20.4	5.0	−2.4

The parameter value in table represents observed value for Mtb-RmlA compared with typical value obtained for well-refined structure at same resolution.

**Table 4:** Summary of docking results of ligands to the Mtb-RmlA model.

Compound Ethambutol	Free energy of binding (kcal/mol)	Docked energy (kcal/mol)	RMSD (Å)
EMB-1	−6.04	−8.88	0.13
EMB-2	−5.82	−8.66	0.54
EMB-3	−5.68	−8.37	0.61
EMB-4	−5.34	−8.02	0.75
EMB-5	−4.92	−7.73	0.98
EMB	−4.90	−7.69	1.54
Natural substrate (Glc-1-P)	−6.01	−8.85	0.19
