# Supplementary material for: Inhibition of Mycobacterium-RmlA by Molecular Modeling, Dynamics Simulation, and Docking
Source: Adv Bioinformatics. 2016 Feb 14;2016:9841250. doi: 10.1155/2016/9841250 (PMC4769735; doi:10.1155/2016/9841250)
Supplement: Supplementary file 1 — With the tertiary structure of Mtb-RmlA of templates retrieved from RCSB-PDB, they have backbone RMS values of Mtb-RmlA-1H5T, Mtb-1H5R, Mtb-1H5S, and Mtb-1IIM which are 0.60 Å, 0.57 Å, 0.65 Å, and 0.61 Å, respectively. [file 9841250.f1.docx]

**Table: Drug likeness properties of 35 EMB lead molecules in the order of priority.**

| **Compound** | **mi**  **Log P** | **TPSA** | **Natoms** | **MW** | **noN** | **nOHNH** | **N**  **Violations** | | **nrotb** | | **Volume** | | |
| --- | --- | --- | --- | --- | --- | --- | --- | --- | --- | --- | --- | --- | --- |
| 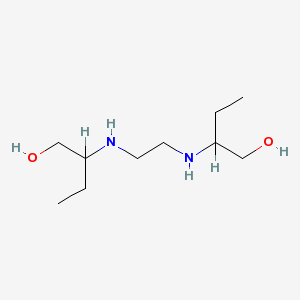EMB1 | 0.431 | 53.516 | 14 | 204.314 | 4 | 3 | 0 | | 9 | | 221.79 | | |
| 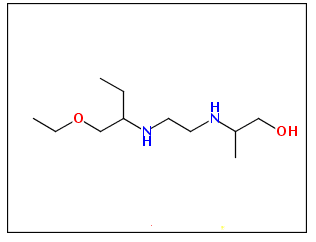EMB2 | 0.807 | 53.516 | 15 | 218.341 | 4 | 3 | 0 | | 10 | | 238.591 | | |
| 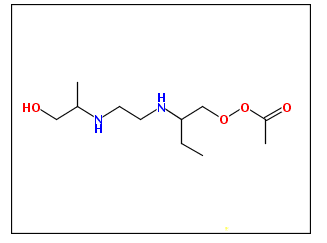EMB3 | 0.477 | 79.821 | 17 | 248.323 | 6 | 3 | 0 | | 11 | | 249.758 | | |
| 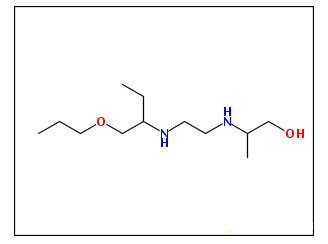EMB4 | 1.309 | 53.516 | 16 | 232.368 | 4 | 3 | 0 | | 11 | | 255.393 | | |
| 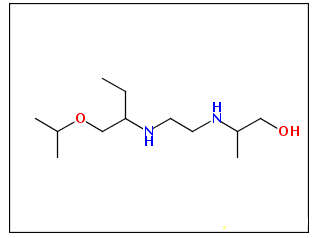EMB5 | 1.17 | 53.516 | 16 | 232.368 | 4 | 3 | 0 | | 10 | | 255.178 | | |
| 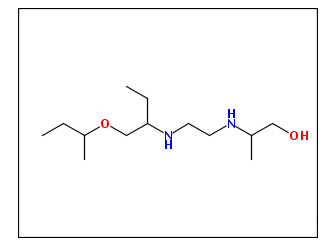EMB6 | 1.672 | 53.516 | 17 | 246.395 | 4 | 3 | 0 | | 10 | | 255.178 | | |
| 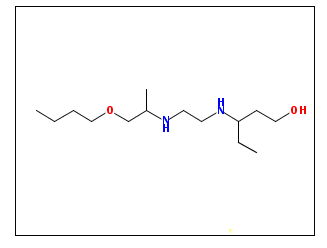EMB7 | 1.869 | 53.516 | 17 | 246.395 | 4 | 3 | 0 | | 12 | | 272.195 | | |
| 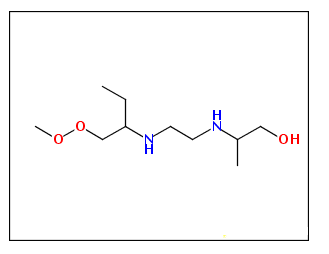EMB8 | 0.39 | 62.75 | 15 | 220.313 | 5 | 3 | 0 | | 10 | | 230.774 | | |
| 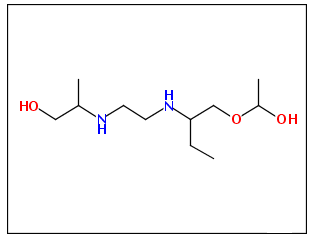EMB9 | 0.149 | 73.744 | 16 | 213.34 | 5 | 4 | 0 | | 10 | | 246.635 | | |
| 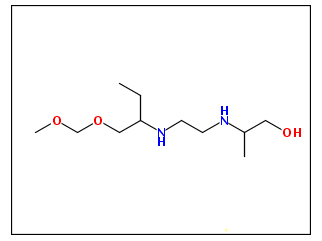EMB10 | 0.402 | 62.75 | 16 | 234.34 | 5 | 3 | 0 | | 11 | | 247.576 | | |
| 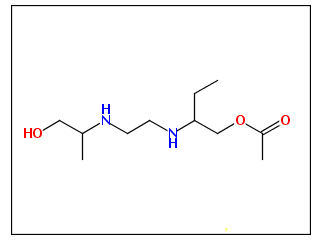EMB11 | 0.518 | 70.587 | 16 | 232.324 | 5 | 3 | 0 | | 10 | | 240.773 | | |
| 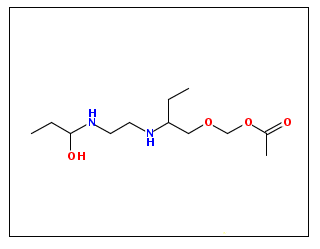EMB12 | -0.024 | 70.587 | 17 | 246.531 | 5 | 3 | 0 | | 11 | | 257.575 | | |
| 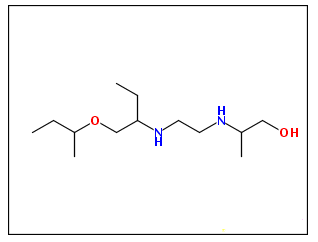EMB13 | -0.72 | 115.418 | 18 | 261.37 | 7 | 7 | 1 | | 12 | | 268.019 | | |
| 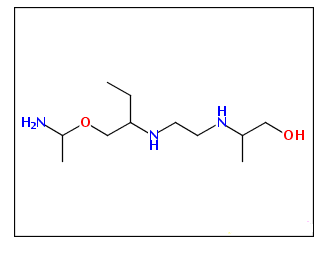EMB14 | 0.192 | 79.539 | 16 | 233.356 | 5 | 5 | 0 | | 10 | | 249.906 | | |
| 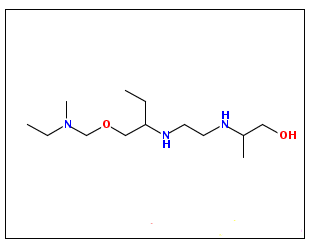EMB15 | 0.824 | 56.754 | 18 | 261.41 | 5 | 8 | 0 | | 12 | | 284.738 | | |
| 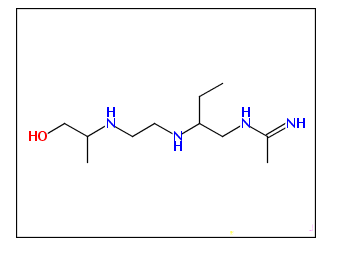EMB16 | 0.286 | 80.161 | 16 | 230.356 | 5 | 5 | 0 | | 10 | | 247.505 | | |
| 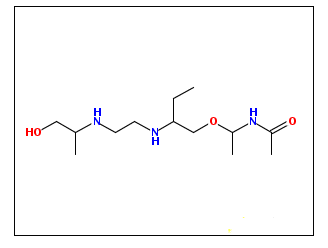EMB17 | -0.073 | 82.164 | 19 | 275.393 | 6 | 4 | 0 | | 11 | | 286.564 | | |
| 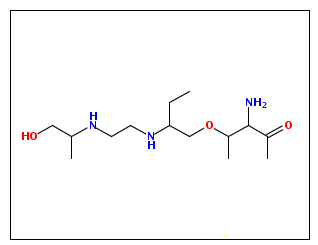EMB18 | -0.797 | 96.61 | 20 | 289.42 | 6 | 5 | | 0 | | 12 | | 302.278 |  |
| 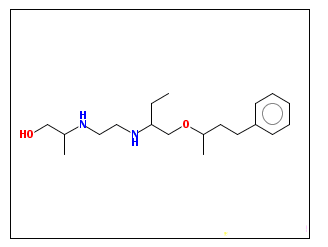EMB19 | 3.116 | 53.516 | 23 | 322.493 | 4 | 3 | | 0 | | 13 | | 343.629 |  |
| 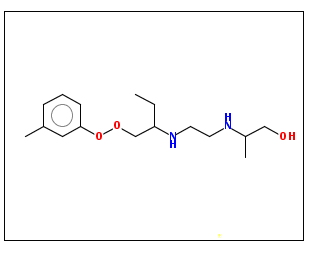EMB20 | 2.45 | 53.516 | 21 | 294.439 | 4 | 3 | | 0 | | 11 | | 310.0 |  |
| 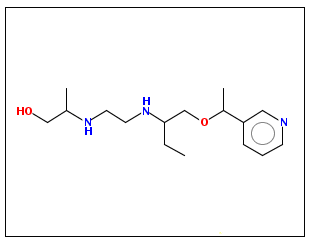EMB21 | 1.152 | 66.408 | 21 | 295.427 | 5 | 3 | | 0 | | 11 | | 305.869 |  |
| 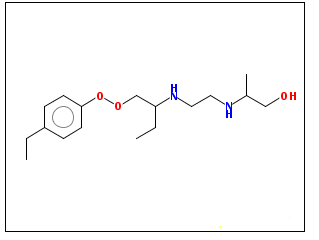EMB22 | 3.002 | 62.75 | 22 | 310.438 | 5 | 3 | | 0 | | 12 | | 318.985 |  |
| 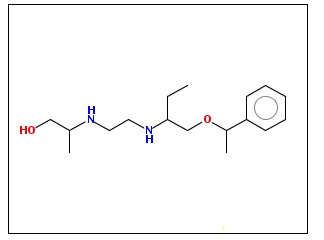EMB23 | 2.389 | 53.516 | 21 | 294.439 | 4 | 3 | | 0 | | 11 | | 310.206 |  |
| 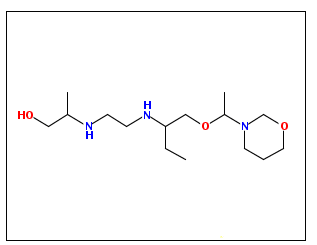EMB24 | 5.113 | 68.571 | 21 | 299.415 | 6 | 3 | | 0 | | 11 | | 304.378 |  |
| 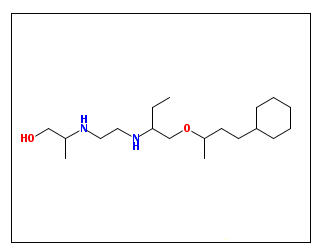EMB25 | 3.806 | 53.516 | 23 | 328.541 | 4 | 3 | | 0 | | 13 | | 362.216 |  |
| 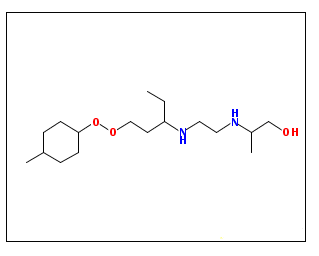EMB26 | 2.726 | 53.516 | 21 | 330.487 | 4 | 3 | | 0 | | 11 | | 328.612 |  |
| 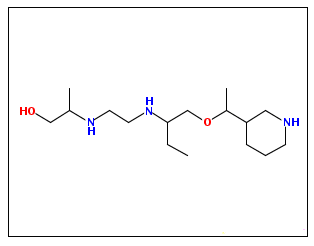EMB27 | 1.719 | 56.744 | 21 | 301.475 | 5 | 3 | | 0 | | 11 | | 324.568 |  |
| 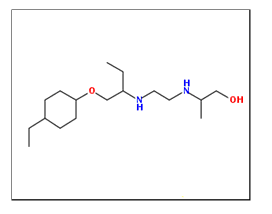EMB28 | 3.028 | 53.516 | 21 | 300.487 | 4 | 3 | | 0 | | 11 | | 328.612 |  |
| 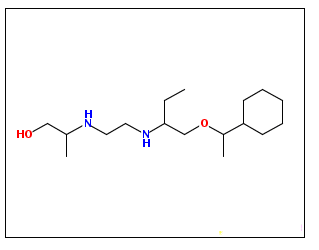EMB29 | 3.082 | 53.516 | 21 | 300.487 | 4 | 3 | | 0 | | 11 | | 328.612 |  |
| 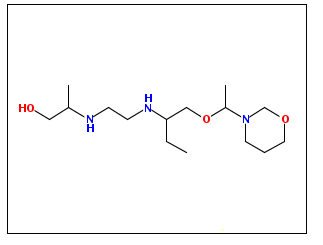EMB30 | 0.915 | 65.988 | 21 | 303.447 | 6 | 3 | | 0 | | 11 | | 316.751 |  |
| 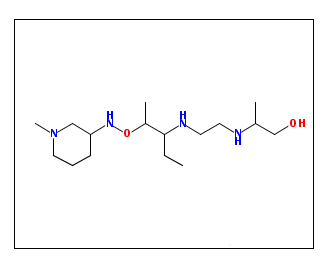EMB31 | 0.34 | 59.992 | 21 | 302.463 | 6 | 3 | | 0 | | 11 | | 320.525 |  |
| 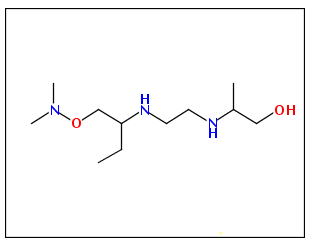EMB32 | 0.435 | 56.754 | 16 | 233.356 | 5 | 3 | | 0 | | 10 | | 278.422 |  |
| 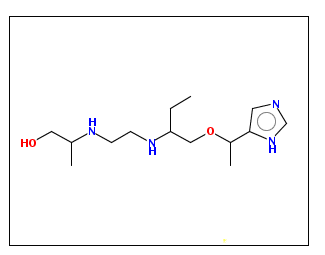EMB33 | 1.83 | 53.516 | 18 | 258.406 | 4 | 3 | | 0 | | 1 | | 278.422 |  |
| 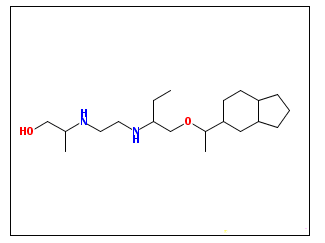EMB34 | 3.486 | 53.516 | 21 | 340.552 | 4 | 3 | | 0 | | 11 | | 368.228 |  |
| 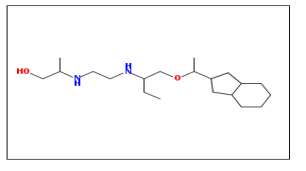EMB35 | 3.72 | 53.516 | 24 | 340.552 | 4 | 3 | | 0 | | 11 | | 368.228 |  |
